# The Diagnosis and Treatment of Quinsy

**Published:** 1909-01-02

**Authors:** 


### Surgery.

### THE DIAGNOSIS AND TREATMENT OF QUINSY.

Quinsy is a popular name for an acute unilateral parenchymatous inflammation of the tonsil. In other words, it is an acute inflammatory condition in which not only the tissues of the tonsil itself are concerned, but the peritonsillar tissues also.

Clinically it is quite unlike a case of follicular tonsillitis: this is generally bilateral, and consists of an acute inflammatory condition of the mucous coat of the tonsillar crypts, so that pus is poured out of their openings.

In both, however, the actual method of causation is somewhat obscure. All that we can say is that as the result of some diminution of the normal resistance of the patient to bacterial invasion the tonsil becomes infected; but it is difficult to distinguish the particular organism which is the *fons et origo* on account of the multitude of them which are normal inhabitants of the buccal cavity: and then, again, certain general infections seem closely allied to infective conditions of the tonsils, particularly rheumatism, scarlet fever, and influenza. Some authorities suppose that the tonsil acts as a filter to all micro-organisms which find their way into the mouth or pharynx, and, if this be true, it is easy to understand that, the moment the resistance of an individual is even momentarily depressed, the tonsil is the first organ to suffer.

### Symptoms and Differential Diagnosis.

In both follicular and parenchymatous tonsillitis the constitutional symptoms are severe. There is malaise, anorexia, and pyrexia, the temperature often rising to 103° F., or even higher. If anything, the general condition is worse in quinsy than it is in follicular tonsillitis. The high temperature helps one to distinguish these cases from diphtheria, for in this a high pyrexia is the exception rather than the rule.

The first symptom which attracts the patient's attention to his condition is pain on swallowing. This often makes its appearance before any of the constitutional symptoms, and its onset should be sufficient warning that the tonsil is probably inflamed. The pain becomes progressively worse, and in a short time the patient usually experiences some difficulty in opening his mouth—a fact which renders the making of an accurate diagnosis a matter of some difficulty if the case be seen then for the first time. But, apart from ankylosis of the temporo-maxillary joint, whether produced by injury or disease, there are not many causes of inability to open the jaw, and they can generally be excluded. The commonest are mumps, tetanus in its early stages, and the eruption of wisdom teeth.

If a view of the inside of the mouth can be obtained a characteristic appearance will be presented. One half of the soft palate will be bulged forwards towards the tongue, and it will be intensely inflamed, bright red, and œdematous; and not only does it bulge forwards, but it is depressed also, so that the arch of the palate is lower on the affected than on the sound side. Those who expect to see an enlarged tonsil will be disappointed; for it must be remembered that although the condition starts in the tonsil, it involves the periarticular structures as well, and the soft palate and anterior pillar of the fauces become so swollen that the tonsil itself, although enlarged, is hidden from view. After a short time pus is formed, and this may find an exit spontaneously by bursting either through the anterior pillar of the fauces or between that and the tonsil. In the former case an acute perforation, discharging pus, will be found.

When this is the state of affairs the diagnosis is simple. The only conditions which can be possibly mistaken for it are a sarcoma or a gumma of the tonsil. But in these growth is slow and comparatively painless, and there is no pyrexia. Again, in quinsy there may be secondary inflammatory changes in the cervical glands. These also become enlarged in sarcoma, but none of the signs of acute inflammation will be found in them. It was, however, the writer's experience on one occasion to open a gumma of the tonsil in mistake for a quinsy; but in this case the patient was also suffering at the time from influenza, which was giving rise to severe constitutional symptoms, and thus rendered the diagnosis a matter of more than ordinary difficulty. Fortunately the patient was none the worse for the treatment.

### Method of Treatment.

The right line of treatment in a quinsy is to open it as soon as it is diagnosed. It does not matter whether pus has already formed or not. If it has not, the condition may be described as one of peritonsillar cellulitis, and incision will give as much relief as opening an abscess. An anæsthetic should never be given. To do so is to court disaster, because in many cases there is also some secondary œdema of the aryteno-epiglottidean folds, and inspiration may become seriously embarrassed. All that is necessary is to cocainise the pharynx thoroughly with a spray some minutes before the operation is to be performed.

It is best to place the patient on a chair and to sit upon a second one directly facing him. The mouth is then opened to the widest possible extent, and the patient's tongue is depressed with a spatula which is held in the surgeon's left hand. In his right he takes a scalpel, the blade of which is wrapped round with strapping up to a quarter of an inch from its point. This is done merely as a guide to enable one to know how deeply the knife has been inserted. The point of the scalpel is then pushed through the anterior pillar of the fauces, the cutting edge turned inwards, and an incision made in a direction upwards and inwards parallel to the fibres of the muscles which form the soft palate. In most cases immediate relief is given by this manœuvre. It need hardly be said that a brisk purge should also be administered. The drugs that appear to do the most good are quinine and sodium salicylate. These may be conveniently combined by giving two grains of quinine salicylate every four hours until the inflammation subsides.

